# Age‐anchored genetic risk for Alzheimer’s disease is associated with markers of cerebrovascular disease, inflammation, and neurodegeneration in older adults without dementia

**DOI:** 10.1002/alz70862_109833

**Published:** 2025-12-23

**Authors:** Rafael V. Lippert, A. Zarina Kraal, Natalie C. Edwards, Giuseppe Tosto, Sandra Barral, Jeffrey D. Pyne, Patrick J. Lao, Adam Brickman

**Affiliations:** ^1^ Columbia University Irving Medical Center, New York, NY USA; ^2^ Taub Institute for Research on Alzheimer’s Disease and the Aging Brain, Vagelos College of Physicians and Surgeons, Columbia University, New York, NY USA; ^3^ Taub Institute for Research on Alzheimer's Disease and the Aging Brain, New York, NY USA; ^4^ Gertrude H. Sergievsky Center, Columbia University Irving Medical Center, New York, NY USA

## Abstract

**Background:**

Cerebrovascular disease (CVD), inflammation, and neurodegeneration increase risk for Alzheimer’s disease (AD). Examination of whether genetic risk for AD is associated with these factors and whether they mediate cognition provides insights into pathways that link genetic susceptibility with clinical outcomes. We examined the associations of an age‐anchored polygenic risk score (cumulative incidence rate; CIR) with markers of CVD, inflammation, and neurodegeneration in unimpaired older adults. We tested whether these factors mediate the association between CIR and cognition.

**Method:**

We included 601 unimpaired participants from ADNI (age=72.6±7.12, 47% women), with available white matter hyperintensity (WMH) volumes, CIR scores, amyloid PET SUVR, and composite cognitive scores; a subset of 92 participants had available plasma GFAP and NfL values. CIR scores represent an age‐anchored and annualized risk for developing AD by combining incidence rates and polygenic hazard scores, calculated from 32 genetic variants. Linear models examined the relationship of CIR scores with WMH, GFAP and NfL concentrations, and cognitive scores. Stratified analysis evaluated the observed associations by amyloid‐beta (Aβ) positivity status. Mediation analyses tested whether WMH and biomarkers mediated the relationship between CIR and cognition, in models adjusted for sex/gender and education.

**Result:**

Higher CIR scores were associated with greater WMH volume (B=4.29; CI[3.14,5.44]; *p* <0.0001), NfL (B=66.68; 95% CI[32.37,101.01]; *p* <0.001), and GFAP (B=390.64;; 95% CI[209.19,572.09]; *p* <0.0001). The association between CIR and WMH was observed in both Aβ+ (B=2.92, CI[1.23,4.61], *p* <0.001) and Aβ‐ (B=5.17; CI[0.20,0.40]; *p* <0.0001) groups.

Higher CIR were associated with lower executive function (B=‐1.946; CI[‐2.92,‐0.98]; *p* <0.0001), memory (B=‐2.947; CI[‐4.03,‐1.87]; *p* <0.0001), and language scores (B=‐2.101; CI[‐3.08,‐1.12]; *p* <0.0001).

White matter hyperintensity volumes mediated the relationship of CIR with executive function (ACME=‐0.26; 95% CI [‐0.50,‐0.05]; *p* =0.028), memory (ACME=‐0.26; 95% CI [‐0.52,‐0.06]; *p* =0.016), and language (ACME=‐0.24; 95% CI [‐0.51,‐0.01]; *p* =0.036). NfL concentration mediated the relationship between CIR and executive function (ACME=‐0.69, CI[‐1.86,‐0.04]; *p* =0.02).

**Conclusion:**

Increased genetic risk for AD in cognitively unimpaired older adults is associated with cerebrovascular burden, higher GFAP and NfL concentrations, and lower cognitive scores. White matter hyperintensities mediate the association between genetic risk for AD and cognition, including in individuals without evidence of amyloid, suggesting a role of cerebrovascular disease in genetic risk for clinical AD.

